# Useful Bicistronic Reporter System for Studying Poly(A) Site-Defining *cis* Elements and Regulation of Alternative Polyadenylation

**DOI:** 10.3390/ijms19010279

**Published:** 2018-01-17

**Authors:** Zhongyuan Deng, Shen Zhang, Shaohua Gu, Xinzhi Ni, Wenxian Zeng, Xianchun Li

**Affiliations:** 1 College of Animal Science and Technology, Northwest A&F University, No. 22 Xinong Road, Yangling 712100, Shaanxi, China; dengzhongyuan@outlook.com; 2 State Key Laboratory for Biology of Plant Diseases and Insect Pests, Institute of Plant Protection, Chinese Academy of Agricultural Sciences, Beijing 100193, China; zhang_shen02@163.com (S.Z.); gushaohua007@163.com (S.G.); 3 United States Department of Agriculture, Agricultural Research Service, Crop Genetics and Breeding Research Unit, Tifton, GA 31793, USA; xinzhi.ni@ars.usda.gov; 4 Department of Entomology and BIO5 Institute, University of Arizona, Tucson, AZ 85721, USA

**Keywords:** alternative polyadenylation, *cis* elements, bicistronic reporter system, human CD47, polyadenylation efficiency, synthetic polyadenylation sites

## Abstract

The link between polyadenylation (pA) and various biological, behavioral, and pathological events of eukaryotes underlines the need to develop in vivo polyadenylation assay methods for characterization of the *cis*-acting elements, *trans*-acting factors and environmental stimuli that affect polyadenylation efficiency and/or relative usage of two alternative polyadenylation (APA) sites. The current protein-based CAT or luciferase reporter systems can measure the polyadenylation efficiency of a single pA site or candidate *cis* element but not the choice of two APA sites. To address this issue, we developed a set of four new bicistronic reporter vectors that harbor either two luciferase or fluorescence protein open reading frames connected with one Internal Ribosome Entry Site (IRES). Transfection of single or dual insertion constructs of these vectors into mammalian cells demonstrated that they could be utilized not only to quantify the strength of a single candidate pA site or *cis* element, but also to accurately measure the relative usage of two APA sites at both the mRNA (qRT-PCR) and protein levels. This represents the first reporter system that can study polyadenylation efficiency of a single pA site or element and regulation of two APA sites at both the mRNA and protein levels.

## 1. Introduction

Polyadenylation is an essential step of gene regulation that directs all biological and behavioral events of eukaryotes [1,2,3]. This transcription-coupled process is initiated by recognition of a pA site in the pre-mRNA, followed by termination of transcription, endonucleolytic cleavage of the pre-mRNA, and addition of a non-templated pA tail [1,2]. It was long held that pA sites are specified primarily by a highly conserved AAUAAA hexamer signal 10–30 bp 5′ to the cleavage site and a more variable U/UG-rich element 15–30 bp 3′ of the cleavage site. However, *in silico* analysis of the pA tail-containing transcripts from human, mouse, freshwater planarian (*Schmidtea mediterrane*), and fruit fly (*Drosophila melanogaster*) actually reveals three types of pA sites: canonical AAUAAA sites (specified by an upstream AAUAAA hexamer and a downstream U-/UG-rich element; 40–49%, depending on the species and data set examined), non-canonical ones (defined by one of the single-base variants of AAUAAA hexamer and a downstream U-/UG-rich element; 25–40%), and AAUAAA-like hexamer-independent ones (with no recognizable AAUAAA-like hexamer and U-/UG-rich element; 13–25%) [4,5,6,7,8,9,10,11]. Which *cis* elements define the 13–25% AAUAAA-like hexamer-independent pA sites remains underexplored.

The three types of pA sites, particularly the weaker non-canonical and AAUAAA-like hexamer-independent pA sites, often also possess up- and/or down-stream auxiliary *cis* elements in addition to their core *cis* elements, which have been studied only in a small number of mRNAs [12]. Among the several characterized auxiliary *cis* elements are the upstream U-rich elements [13], UGUA element [14,15], and US1A [12] as well as the downstream G-rich elements [16] and DS1A [12]. These auxiliary *cis* elements facilitate polyadenylation by serving as additional anchors for the polyadenylation machinery, or by recruiting specific *trans*-acting protein factors [1,2,3,17,18,19]. Many more auxiliary *cis* elements are yet to be identified.

Due to the presence of two or more alternative pA sites, a large proportion of eukaryotic genes (e.g., ~70% of human genes) undergoes alternative polyadenylation (APA), producing several mRNA isoforms with variable length of 3′ coding sequence (coding sequence APA) and/or 3′ untranslated regions (3′ UTR; UTR APA) [9,20,21,22,23]. This can change the 3’ coding sequence and/or microRNAs binding sites of all the mRNA isoforms of each gene and thus affect their function, stability, exportation, localization, and translation efficiency [9,20,21,22,23,24]. The proximal pA sites are often non-canonical AAUAAA variant sites and therefore generally weaker, whereas the distal pA sites are usually stronger canonical AAUAAA sites [3,4,6,9]. Selective usage of two or more APA sites is regulated by the “strength” of the core and auxiliary *cis*-acting elements defining each APA site and their positions, the isoform and abundance or activity of the core 3’-processing protein factors, and of other protein factors such as transcription factors, splicing factors, RNA-binding proteins that enhance or repress assembly of the core 3’-processing factors to each APA site, and variations in nucleosome organization and epigenetic marks around each APA site [3,6,25]. Selection of APA sites is also influenced by extracellular stimuli that induce differential expression of the core 3’-processing factors, RNA-binding proteins, splicing factors, and transcription factors [3,9,26,27,28,29]. As in the case of *cis* elements, exactly how and which *trans*-acting factors and environmental stimuli impact polyadenylation efficiency and selective/relative usage of APA sites are yet to be elucidated.

Characterization of the core *cis* elements defining the AAUAAA-like hexamer-independent pA sites as well as of the additional auxiliary *cis* elements, *trans*-acting protein factors, and extracellular stimuli that affect the strength of pA sites and the choice of APA sites necessitates development of in vivo polyadenylation assay methods for quantifying polyadenylation efficiency and relative usage of APA sites. The protein-based CAT [30,31] or luciferase [12,32,33,34,35] reporter system has been adapted to measure the polyadenylation efficiency of a pA cassette (the genomic sequence fragment surrounding a given pA site, which contains the core and accessary *cis* elements of the pA site) [30,31,32] or a bioinformatically identified novel candidate core or auxiliary element [12], but is unable to quantify the relative usage of two APA sites. While the luciferase reporter plasmid pPASPORT used by Yao et al. [35] yields a bicistronic mRNA containing two reporter genes (*Rluc* and *Fluc*), it has only one multiple cloning site and thus still cannot be used to study the relative usage of two APA sites. The RNA-based in vivo tandem polyadenylation assay relying on PhosphorImager quantification of RNase protected RNA bands extracted from the cells transfected with the pCßS-proximal pA-distal pA-BGH pA vector construct [12,36] can measure the polyadenylation efficiency of a single pA site or candidate *cis* element and the relative usage of two APA sites, but has to generate and use α-^32^P-radiolabeled pA site-specific antisense RNA probe.

In this study, we developed four new bicistronic reporter vectors that harbor either two fluorescence protein ORFs (pCMV-DsRed-MCS-IRES-EGFP-SV40 pA and pTK-DsRed-MCS-IRES-EGFP-SV40 pA) or two luciferase ORFs (pSV40-hRluc-MCS-IRES-hluc-SV40 pA and pTK-hRluc-MCS-IRES-hluc-SV40 pA) connected with a multiple cloning site plus the Internal Ribosome Entry Site (IRES). The two vectors that use the CMV promoter (pCMV) and SV40 promoter (pSV40) are used to determine the strength of a pA site or candidate element and the relative usage of two APA sites in mammalian cells such as human HEK293 and Hela cells, whereas the two vectors that use TK promoter (pTK) are utilized to measure the polyadenylation efficiency of a single pA site or candidate *cis* element and the relative usage of two APA sites in insect cell line such as *Drosophila melanogaster* S2 cells, *Helicoverpa zea* fatbody cells and *Bombyx mori* BM-N cells. Our test experiments with the recombinant constructs containing a single pA site, a *cis* elements or two pA sites demonstrated that these bicistronic reporter vectors could be readily used to characterize *cis* elements, *trans*-acting factors, and regulation of APA.

## 2. Results

### 2.1. Construction and Working Principle of the Bicistronic Reporter System

**Figure 1:**
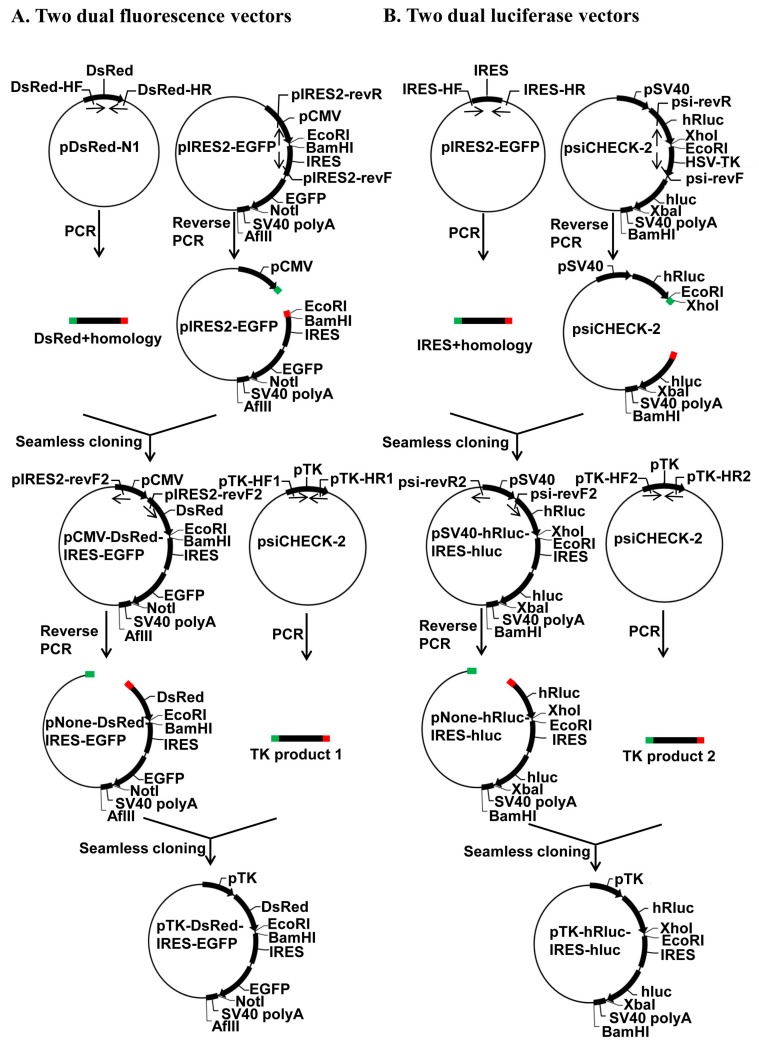
Schematic diagram for construction of the bicistronic reporter system. (**A**) Construction of 2 dual fluorescence reporter vectors pCMV-DsRed-MCS-IRES-EGFP-SV40 pA (for use in mammalian cells) and pTK-DsRed-MCS-IRES-EGFP-SV40 pA (for use in insect cells); (**B**) Construction of 2 dual luciferase reporter vectors pSV40-hRluc-MCS-IRES-hluc-SV40 pA (for use in mammalian cells) and pTK-hRluc-MCS-IRES-hluc-SV40 pA (for use in insect cells). pCMV = CMV promoter, pTK = HSV-TK promoter, pSV40 = SV40 promoter, SV40 pA = SV40 polyadenylation site sequence (Poly(A)), DsRed = *Discosoma* sp. Red Fluorescent Protein, EGFP = Enhanced Green Fluorescent Protein, hluc = humanized firefly luciferase, hRluc = humanized *renilla* luciferase, IRES = Internal Ribosome Entry Site. The terminal 15–20 bp homology sequences shared between an insert and the corresponding linearized plasmid are essential for seamless cloning, and thus are colored in red and green, respectively.

Sequencing of the positive clones verified that we successfully generated a bicistronic reporter system composed of two dual fluorescence (pCMV-DsRed-MCS-IRES-EGFP-SV40 pA and pTK-DsRed-MCS-IRES-EGFP-SV40 pA) (Figure 1A) and two dual luciferase (pSV40-hRluc-MCS-IRES-hluc-SV40 pA and pTK-hRluc-MCS-IRES-hluc-SV40 pA) (Figure 1B) reporter vectors by the seamless cloning strategy. The four vectors differ in their reporter proteins (fluorescence protein DsRed plus EGFP vs. humanized firefly (hluc) plus *renilla* (hRluc) luciferase) and promoters (pCMV or pSV40 vs. pHSV-TK) that drive the transcription of the reporter genes. The two vectors with a pCMV (pCMV-DsRed-MCS-IRES-EGFP-SV40 pA) or pSV40 (pSV40-hRluc-MCS-IRES-hluc-SV40 pA) promoter are compatible with mammalian cell lines such as human HEK293 and Hela cells, whereas the two vectors with a pHSV-TK promoter are used to drive the expression of the reporter genes in insect cell lines such as *Drosophila melanogaster* S2 cells, *Spodoptera frugiperda* Sf9, *Helicoverpa zea* fatbody cells, and *Bombyx mori* BM-N cells. The fluorescence proteins expressed from the two dual fluorescence vectors can be intuitively and quantitatively detected by a fluorescence microscope or cell flow cytometry, whereas the luciferase expressed from the two dual luciferase vectors can be quantitatively measured by a luminometer.

**Figure 2:**
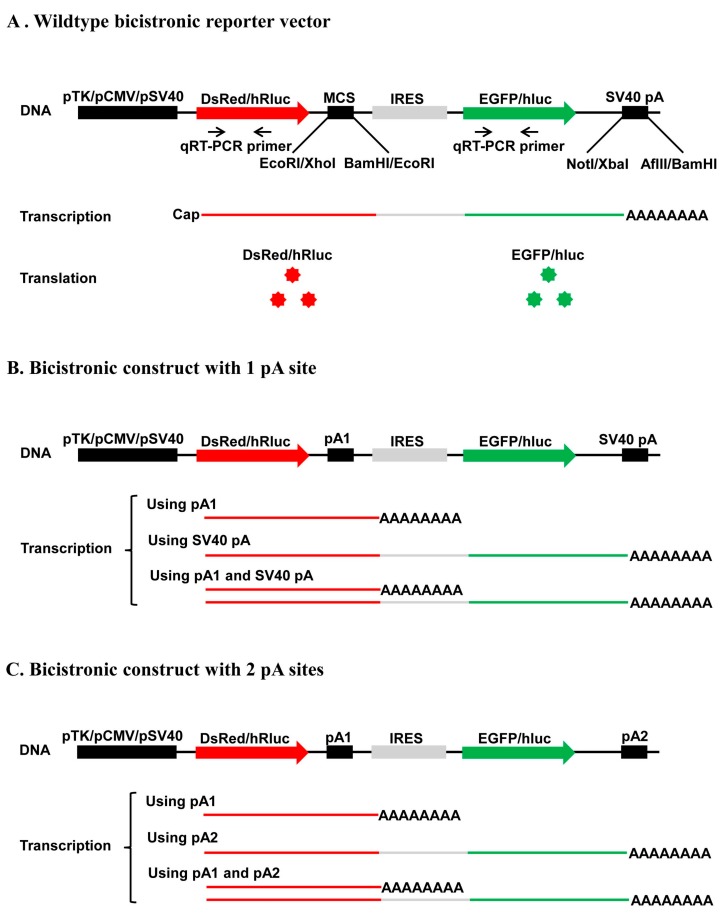
Diagram of the working principle of the bicistronic reporter system. (**A**) Wildtype bicistronic reporter vector is supposed to transcribe into a bicistronic mRNA possessing 1 cap, 2 ORFs (DsRed and EGFP for the 2 fluorescence wildtype vectors and hluc and hRluc for the 2 luciferase wildtype vectors), and 1 pA tail when transfected into mammalian or insect cells. Translation of the 1st (DsRed or hRluc) and 2nd ORFs (EGFP or hluc) is initiated at the normal 5′ cap and the IRES, respectively; (**B**) Bicistronic construct with 1 pA site may transcribe into (1) a monocistronic mRNA containing only the 1st ORF; (2) a bicistronic mRNA possessing both ORFs as the wildtype vectors; and (3) both of the above, depending on the polyadenylation capability of the inserted candidate pA site relative to that of the SV40 pA site; (**C**) Bicistronic construct with 2 pA sites may also transcribe into (1) a monocistronic mRNA containing only the 1st ORF; (2) a bicistronic mRNA possessing both ORFs as the wildtype vectors; and (3) both of the above, depending on the relative polyadenylation efficiency of the two inserted candidate pA sites.

Regardless of the differences in their reporter proteins and promoters, the four vectors share a common structure framework consisting of one promoter, follow by 1st ORF, two restriction enzyme sites, one IRES element, 2nd ORF, one restriction enzyme site, one pA site (SV40 pA), and another restriction enzyme site (Figure 2A). Theoretically, this common structure framework should allow them to be transcribed into a bicistronic mRNA possessing one pA tail and two ORFs connected by an IRES element when transfected into mammalian or insect cells. The insertion of one IRES immediately upstream of the 2nd ORF should allow translation of both ORFs, with the 1st ORF initiated at the normal 5′ cap, and the second at the IRES (Figure 2A). Thus, the expression of both ORFs can be quantitatively analyzed both at the mRNA level by qRT-PCR and at the protein level by fluorescence microscopy/flow cytometry or luciferase assay.

The common structure framework allows insertion of one candidate pA site into the 1st two restriction enzyme sites, i.e., *EcoRI* and *BamHI* sites in the two dual fluorescence vectors or *XhoI* and *EcoRI* sites in the two dual luciferase vectors, producing a recombinant construct (Figure 2B). Depending on the polyadenylation capability of the inserted candidate pA site relative to that of the SV40 pA site, the recombinant construct may transcribe into (1) a monocistronic mRNA containing only the 1st ORF; (2) a bicistronic mRNA possessing both ORFs as the wildtype vectors do; and (3) both of the above (Figure 2B). Moreover, the common structure framework also allows simultaneous cloning of one candidate pA site into the 1st two restriction enzyme sites and another to replace the SV40 pA site through the two restriction enzyme sites immediately up- and down-stream of the SV40 pA site, i.e., *NotI* and *AflII* sites in the two dual fluorescence vectors or *XbaI* and *BamHI* sites in the two dual luciferase vectors (Figure 2C). Likewise, depending on the relative polyadenylation efficiency of the two inserted candidate pA sites, the resulted recombinant construct may transcribe into (1) a monocistronic mRNA containing only the 1st ORF; (2) a bicistronic mRNA possessing both ORFs; and (3) both of the above (Figure 2C). Analyses of both ORFs at the mRNA and/or protein level can reveal what mRNAs are produced, the polyadenylation efficiency of the single pA sites tested, and the relative usage/strength of the two APA sites tested.

### 2.2. Characterization of Putative pA Sites with the Bicistronic Reporter System

**Figure 3:**
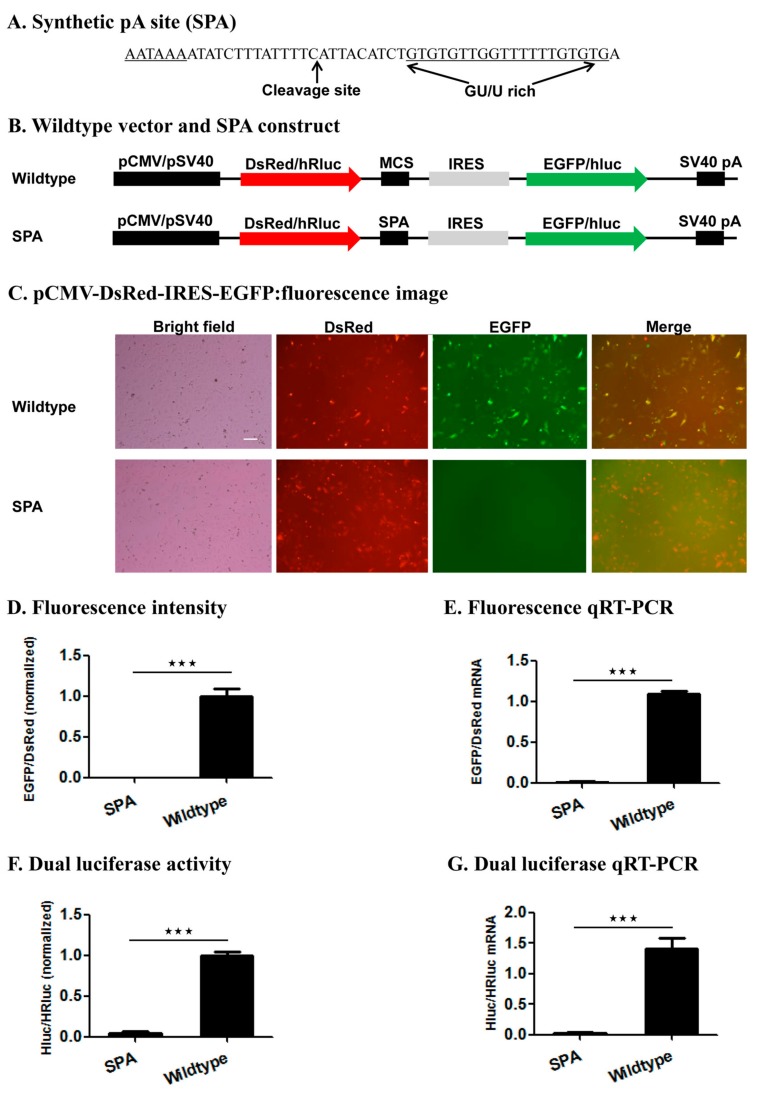
Impact of inserting a synthetic pA (SPA) site on the relative expression of the two luciferase or fluorescence reporter genes. (**A**) Synthetic pA site (SPA); (**B**) Wildtype vector and SPA construct; (**C**) pCMV-DsRed-IRES-EGFP: fluorescence image. Shown here is a representative Hela cell image of 3 independent transfections per construct with a scale bar of 20 μm; (**D**) Fluorescence intensity; (**E**) Fluorescence qRT-PCR; (**F**) pSV40-hRluc-IRES-hluc: dual luciferase activity; (**G**) Dual luciferase qRT-PCR. The data and error bars in (**D**–**G**) represent the means and standard errors of at least three independent transfections (biological replicates) for each wildtype vector or SPA construct. Extremely significant differences between the wildtype vector and SPA construct are indicated by three stars (***, *p* < 0.001).

To test whether the bicistronic reporter system can be used to assess the polyadenylation capacity of a candidate pA site, we cloned a known synthetic pA site (SPA) [35] of 49 bp (Figure 3A) into the first two restriction enzyme sites of the wildtype vectors pCMV-DsRed-MCS-IRES-EGFP-SV40 pA and pSV40-hRluc-MCS-IRES-hluc-SV40 pA, respectively (Figure 3B). The 49 bp pA site contains a 5′ AAUAAA hexamer, followed by a 22-bp spacer sequence possessing a CA cleavage site, and a 21-bp downstream UG/U-rich element (Figure 3A), and was previously confirmed as a strong pA site [37]. Microscopic fluorescence imaging and qRT-PCR analysis showed that Hela cells transfected with the wildtype vector pCMV-DsRed-MCS-IRES-EGFP-SV40 pA, as expected in Figure 2A, expressed both DsRed and EGFP proteins (Figure 3C) and an equal amount of *DsRed* and *EGFP* transcripts (Figure 3E). By contrast, Hela cells transfected with the SPA-containing fluorescence construct produced a much higher amount of DsRed transcript and protein but little or no EGFP transcript and protein (Figure 3D,E). Likewise, Hela cells transfected with the SPA-containing luciferase construct had hluc (2nd ORF)/hRluc (1st ORF) mRNA (0.02028) and activity (0.04862) ratios of 69.23 and 20.57 times smaller than those (1.404 and 1.0) of Hela cells transfected with the wildtype pSV40-hRluc-MCS-IRES-hluc-SV40 pA vector (Figure 3F,G). Both the fluorescence and luciferase results prove that insertion of the SPA immediately downstream of the 1st ORF resulted in transcription of greater amounts of monocistronic mRNA possessing only the 1st ORF but little bicistronic mRNA, consistent with the functionality of the SPA [37].

### 2.3. Characterization of cis-Acting Elements with the Bicistronic Reporter System

**Figure 4:**
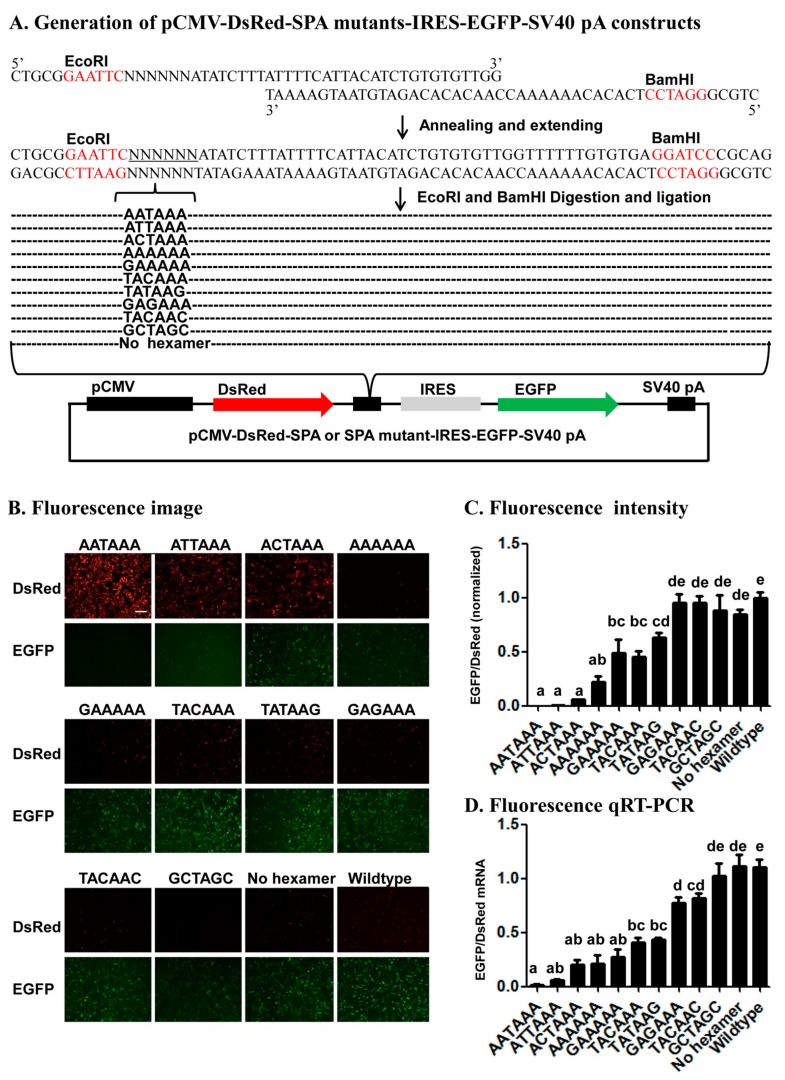
Impact of inserting SPA mutants with different hexamer variants on the relative expression of the two fluorescence reporter genes. (**A**) Generation of pCMV-DsRed-SPA mutants-IRES-EGFP-SV40 pA constructs; (**B**) Fluorescence image. Showed here is a representative HEK293 cell image of 3 independent transfections per construct with a scale bar of 50 μm; (**C**) Fluorescence intensity; (**D**) Fluorescence qRT-PCR. The data and error bars in (**C**,**D**) represent the means and standard errors of at least three independent transfections for each wildtype vector or SPA mutant construct. Values sharing the same letter are not significantly different at *p* < 0.05 (Tukey’s HSD tests).

In order to test if the bicistronic reporter system can be used to identity core or accessary *cis*-acting elements, we ficrst annealed and extended one common reverse oligo (Red-GFP-PAS-R) with five forward oligos that contain AATAAA (Red-GFP-PAS-wild-F), ANTAAA (Red-GFP-PAS-mut1-F), NANAAN (Red-GFP-PAS-mut3-F), NNNNNN (Red-GFP-PAS-rad-F), or no hexamer (Red-GFP-PAS-del-F) (Table S1) to form five different double-stranded SPA fragments. Subcloning of the 5 SPA fragments into the pCMV-DsRed-MCS-IRES-EGFP-SV40 pA vector through its *EcoRI* and *BamHI* sites (Figure 4A) yielded a total of 11 pCMV-DsRed-SPA-IRES-EGFP-SV40 pA constructs that contained AATAAA (from Red-GFP-PAS-wild-F), no hexamer (from Red-GFP-PAS-del-F), 1-base (ATTAAA, ACTAAA and AAAAAA from Red-GFP-PAS-mut1-F), 2-base (GAAAAA, TACAAA, TATAAG and GAGAAA from Red-GFP-PAS-mut3-F), 3-base (TACAAC from Red-GFP-PAS-rad-F), or 4-base (GCTAGC from Red-GFP-PAS-rad-F) variants of the canonical AAUAAA hexamer (Figure 4A).

Transfection of the above 11 SPA constructs into HEK293 cells and subsequent detection of the relative expression of DsRed vs. EGFP at the protein level by microscopic fluorescence imaging (Figure 4B), Image J quantification (Figure 4C) and flow cytometry (Figure S1) and at the mRNA level by qRT-PCR (Figure 4D) showed that the construct with a canonical AAUAAA hexamer had a zero or near-zero mRNA and protein ratio of EGFP/DsRed, whereas the SPA constructs with a AAUAAA variant of ≥3-base substitutions (TACAAC, GCTAGC) or 2 A or T to G changes (GAGAAA) exhibited the similar high mRNA and protein ratios of EGFP/DsRed with the wildtype vector and the SPA construct with no hexamer. The other six SPA constructs with an AAUAAA variant of 1-base (ATTAAA, ACTAAA, AAAAAA) or 2-base (GAAAAA, TACAAA and TATAAG) substitutions displayed a ratio of between the above two opposite ends (Figure 4B–D and Figure S1). Overall, as the number of base changes increased, the mRNA (Figure 4D) and protein (Figure 4B,C and Figure S1) expression levels of DsRed gradually reduced while those of EGFP elevated. When the number of base changes was the same, substitution of A or U to G or C and substitution at position 3 of the AAUAAA hexamer tended to have a higher ratio of EGFP/DsRed than substitution of A (or U) to U (or A) (e.g., ACTAAA vs. ATTAAA, GAAAAA vs. AAAAAA, GAGAAA vs. TACAAA) and substitution at the other positions (e.g., AAAAAA vs. ATTAAA, GAGAAA vs. TATAAG) (Figure 4B–D and Figure S1).

**Figure 5:**
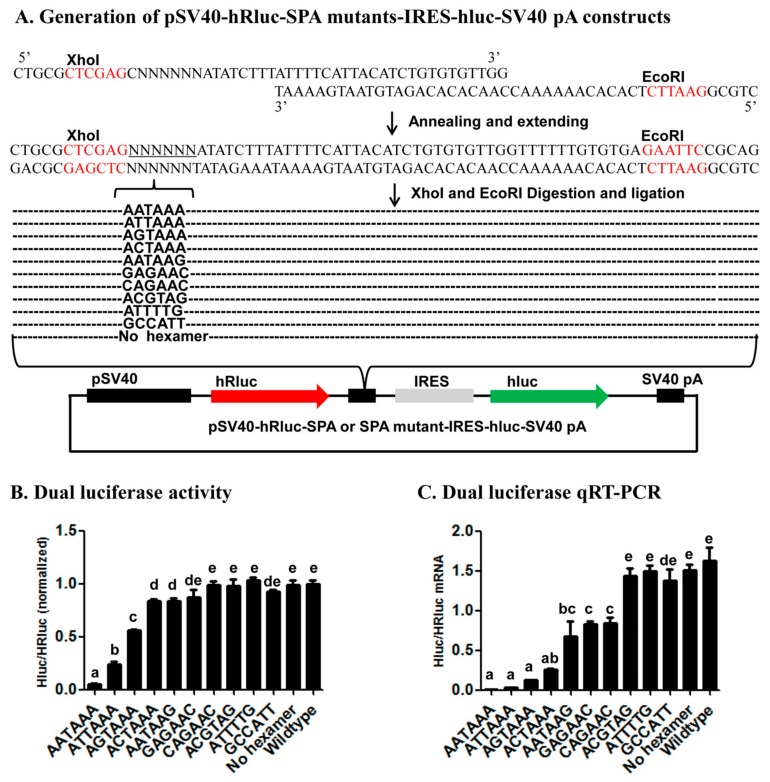
Effects of inserting SPA mutants with different hexamer variants on the relative expression of the two luciferase reporter genes. (**A**) Generation of pSV40-hRluc-SPA mutants-IRES-hluc-SV40 pA constructs; (**B**) Dual luciferase activity; (**C**) Dual luciferase qRT-PCR. The data and error bars in (**B**,**C**) represent the means and standard errors of at least three independent transfections for each wildtype vector or SPA mutant construct. Values sharing the same letter are not significantly different at *p* < 0.05 (Tukey’s HSD tests).

To test if the dual luciferase vector of the bicistronic reporter system can also be used to identity core or accessary *cis*-acting elements, we annealed and extended one common reverse oligo (PSI-R) with five forward oligos that contain AATAAA (PSI-wild-F), ANTAAA (PSI-mut1-F), NANAAN (PSI-mut3-F), NNNNNN (PSI-rad-F), or no hexamer (PSI-del-F) (Table S1) to form five different double-stranded SPA fragments to be inserted into the luciferase vector pSV40-hRluc-MCS-IRES-hluc-SV40 pA through its *XhoI* and *EcoRI* sites (Figure 5A). Sequencing of the resulted positive clones identified one AATAAA (from PSI-wild-F) construct, one no hexamer (from PSI-del-F) construct, four 1-base variant constructs (ATTAAA, ACTAAA and AGTAAA from PSI-mut1-F and AATAAG from PSI-mut3-F), two 3-base variant constructs (GAGAAC and CAGAAC from PSI-mut3-F), two 4-base variant constructs (ACGTAG and ATTTTG from PSI-rad-F) and one 5-base variant construct (GCCATT from PSI-rad-F). Dual luciferase reporter assay and qRT-PCR analysis showed that the AAUAAA SPA construct had near-zero mRNA and protein ratios of hluc/hRluc, whereas the wildtype vector and no hexamer SPA construct produced the highest mRNA and protein ratios of hluc/hRluc (Figure 5B,C). The mRNA and protein ratios of the other nine AAUAAA variant SPA constructs fell somewhere between the two extreme ends, depending on the number (the more the base change, the higher the ratios), position (highest ratios in position 2, followed by position 1 and the other positions) and nature (higher ratios in A or U to C or G substitutions than A (or U) to U (or A) substitutions) of their base changes (Figure 5B,C).

### 2.4. Study of APA with the Bicistronic Reporter System

**Figure 6:**
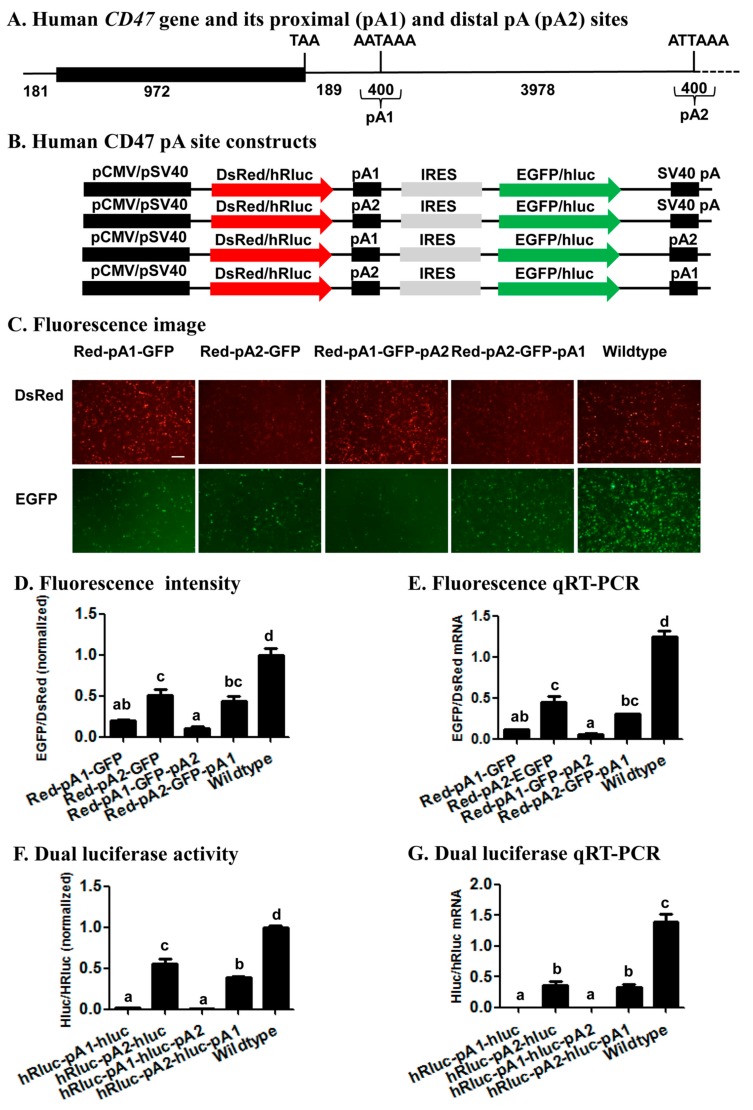
The relative strength of two alternative APA sites from the human CD47 gene. (**A**) Human CD47 gene and its proximal (pA1) and distal pA (pA2) sites; (**B**) Human CD47 pA site constructs; (**C**) Fluorescence image. Showed here is a representative HEK293 cell image of 3 independent transfections per construct with a scale bar of 50 μm; (**D**) Fluorescence intensity; (**E**) Fluorescence qRT-PCR; (**F**) Dual luciferase activity; (**G**) Dual luciferase qRT-PCR. The data and error bars in (**D**–**G**) represent the means and standard errors of at least three independent transfections for each CD47 pA site construct. Values sharing the same letter are not significantly different at *p* < 0.05 (Tukey’s HSD tests).

To test whether the bicistronic reporter system can be utilized to study the relative usage of two APA sites from the same gene, we generated four fluorescence CD47 pA site constructs and four luciferase CD47 pA site constructs by inserting its proximal (pA1), distal (pA2), or both pA sites (200 bp up- and downstream of each cleavage site; see Figure 6A and Figure S2) into the wildtype fluorescence vector pCMV-DsRed-MCS-IRES-EGFP-SV40 pA and the wildtype luciferase vector pSV40-hRluc-MCS-IRES-hluc-SV40 pA, respectively (Figure 6B). When only one CD47 pA site was inserted between DsRed or hRluc and IRES in the two wildtype vectors, the proximal pA site (pA1) had protein and mRNA ratios of EGFP/DsRed (Figure 6C–E) or hluc/Rhluc (Figure 6F,G) significantly smaller than those of the distal pA site (pA2). When both of the CD47 pA sites were inserted into the two wildtype vectors, the two pA1-pA2 constructs (pCMV-DsRed-CD47 pA1-IRES-EGFP-CD47 pA2 and pSV40-hRluc-CD47 pA1-IRES-hluc-CD47 pA2) exhibited significantly smaller protein and mRNA ratios of EGFP/DsRed (Figure 6C–E) or hluc/Rhluc (Figure 6F,G) than those of the two pA2-pA1 constructs (pCMV-DsRed-CD47 pA2-IRES-EGFP-CD47 pA1 and pSV40-hRluc-CD47 pA2-IRES-hluc-CD47 pA1). The EGFP/DsRed or hluc/Rhluc mRNA and protein ratios of all the four CD47 pA site constructs were significantly smaller than those of the wildtype vectors (Figure 6D–G).

## 3. Discussion

*In silico* analysis of ever-increasing transcriptome databases enables bioinformatic identification of putative pA sites, APA sites, and *cis*-acting elements [4,5,6,7,8,9,10,11,12]. Functional characterization of these putative pA sites, APA sites and *cis*-acting elements as well as of the candidate *trans*-acting factors and extracellular stimuli that regulate pA site strength and APA underlines the need for development of in vivo polyadenylation assay methods. In this study, we developed a new bicistronic reporter system composed of two fluorescence vectors and two luciferase vectors that have a common structure frame of 1 promoter + 1st ORF + 2 restriction enzyme sites + 1 IRES element + 2nd ORF + 1 restriction enzyme site + 1 pA site (SV40 pA) and another restriction enzyme site (Figure 2A). This common structure frame allows insertion of one candidate pA site or *cis*-acting element downstream of the 1st ORF and replacement of the SV40 pA with another candidate pA site or *cis-*acting element downstream of the 2nd ORF simultaneously. As a result, this bicistronic reporter system can be utilized not only to quantify the strength of a single candidate pA site or *cis* element, but also to accurately measure the relative usage of two APA sites at both the mRNA (qRT-PCR) and protein levels. By contrast, although the luciferase reporter plasmid pPASPORT used by Yao et al. [35] has two luciferase reporter gene ORFs (*Rluc* and *Fluc*) connected with one IRES element, it cannot be used to study the relative usage of two APA sites because it does not enable simultaneous insertion of two candidate pA sites. Transfection of any one of the four wildtype vectors we developed here into mammalian or insect cells is expected to produce a bicistronic mRNA with an equal molar ratio of *DsRed* (or *hRluc*) and *EGFP* (or *hluc*) ORFs. Consistent with this expectation, we observed an *EGFP*/*DsRed* or *hluc*/*hRluc* mRNA ratio of 1–1.5 (Figure 3E,G). On the other hand, the wildtype luciferase vector yielded a hluc/hRluc activity (i.e., protein level) ratio of about 0.08 before normalization, which certainly can be attributed to the much lower efficiency of IRES-dependent translation than that of cap-dependent translation [38,39]. That the wildtype fluorescence vector yielded an EGFP/DsRed fluorescence intensity (i.e., protein level) ratio of 11.31 before normalization is consistent with the fact that DsRed is only approximately 10% as bright as EGFP [40].

We tested this reporter system by examining the impact of inserting the wildtype SPA (Figure 3) [37] or mutant SPA with a 1–5 base variants of the canonical AAUAAA hexamer element or no hexamer (Figure 4, Figure 5 and Figure S1) immediately downstream of the 1st ORF on the relative expression of the two ORFs (EGFP/DsRed in the fluorescence vector and hluc/Rhluc in the luciferase vector) at both the mRNA and protein levels. Consistent with the functionality of the SPA [37], the wildtype SPA-containing constructs produced huge amounts of monocistronic mRNA possessing only the 1st ORF but little bicistronic mRNAs, whereas both the wildtype fluorescence and luciferase vector were transcribed into bicistronic mRNAs containing both ORFs. This demonstrates the utility of this reporter system for determining whether a candidate pA site is a true functional pA site or not. If a candidate pA site does not change the relative expression of the two ORFs in contrast to the wildtype vector, one would conclude that the candidate pA site is not a true pA site.

Several lines of evidence from the 22 mutant SPA constructs (11 in the fluorescence vector and 11 in the luciferase vector) (Figure 4, Figure 5 and Figure S1) demonstrate that the bicistronic reporter system is a reliable in vivo polyadenylation assay method for characterization of *cis*-acting elements. First, the fact that the relative expression of the two ORFs varied with the number, position, and nature of base changes in each of the 22 mutant SPA constructs suggests that this reporter system is sensitive enough to identify both strong and weak elements that specify pA sites. Second, consistent with the previous bioinformatic studies [4,6,7,10] that ranked the AAUAAA-like hexamers we tested here in the occurring frequency of AAUAAA > AUUAAA > AGUAAA > CAUAAA > AATAAG, this study ranked these hexamers in the polyadenylation efficiency of AAUAAA > AUUAAA > AGUAAA > CAUAAA > AATAAG. Third, early in vitro polyadenylation [41] ranked the polyadenylation efficiency of the hexamers we tested here in the descending order of AAUAAA > AUUAAA > AGUAAA > CAUAAA > ACUAAA > AAAAAA, which is exactly the same order inferred from our data (Figure 4 and Figure 5). Fourth, our finding of position 2 and then 1 were more tolerant to base change than position 3 is matched to the position-nucleotide frequency of the hexamer found in humans [10]. These confirm that the reporter system is a reliable method for characterization of candidate *cis*-acing elements.

Previous reports have demonstrated that the human *CD47* gene was regulated by 3’ UTR APA [24,42], yielding two transcripts that encode the same protein localized to the endoplasmic reticulum when its proximal pA site is used, or the plasma membrane when its distal pA site is used [24]. The relative usage of the two CD47 pA sites revealed with our bicistronic reporter system (Figure 6) suggests that the CD47 proximal pA site (pA1 in Figure 6) is a much stronger pA site than the CD47 distal pA site (pA2), regardless of whether it is placed up- or downstream of the distal pA site. This conclusion not only matches well with the finding of the canonical AAUAAA in the proximal site and of its 1-base variant AUUAAA in the distal site (Figure S2), but is also consistent with the relative endogenous usage of the two pA sites in seven different human cell lines including Hela, HEK293, U2OS, MCF7, NTERA2, B-LCL, and Toledo (Figure 1b,c in Berkovits and Mayr’s research [24]). Such a perfect correlation among the relative strength of two APA sites determined by our bicistronic reporter system, the *cis*-acing elements defining the two APA sits, and the endogenous usage frequency of the two APA sites, validates the utility of the reporter system for study of APA regulation. Specifically, this system can be utilized to identify *trans*-acting proteins or noncoding RNAs and environmental stimuli that affect/regulate the relative usage of two APA sites.

## 4. Materials and Methods

### 4.1. Construction of Four Bicistronic Reporter Vectors

As shown in Figure 1, we constructed two dual fluorescence vectors (pCMV-DsRed-MCS-IRES-EGFP-SV40 pA and pTK-DsRed-MCS-IRES-EGFP-SV40 pA) and two dual luciferase vectors (pSV40-hRluc-MCS-IRES-hluc-SV40 pA and pTK-hRluc-MCS-IRES-hluc-SV40 pA) using the seamless cloning kit (Taihe Biotechnology, Beijing, China). We PCR-amplified the DsRed ORF from the pDsRed1-N1 plasmid (Clontech, Mountain View, CA, USA) with the primers DsRed-homology F and DsRed-homology R (Table S1; abbreviated as DsRed-HF and DsRed-HR in Figure 1A), the IRES element from the pIRES2-GFP plasmid (Clontech) with the primers IRES-homology F and IRES-homology R (Table S1; IRES-HF and IRES-HR in Figure 1B), the HSV-TK promoter (pTK) from psiCHECK-2 (Clontech) with the primer pair pTK-homology F1 and pTK-homology R1 (Table S1; pTK-HF1 and pTK-HR1 for construction of pTK-DsRed-MCS-IRES-EGFP-SV40 pA in Figure 1A) or the primer pair pTK-homology F2 and pTK-homology R2 (Table S1; pTH-HF2 and pTK-HR2 for construction of pTK-hRluc-MCS-IRES-hluc-SV40 pA in Figure 1B). We then conducted 2 reverse PCR to linearize the pIRES2-EGFP plasmid using the primer pair pIRES2-EGFP-reverse F and pIRES2-EGFP-reverse R (Table S1; pIRES2-revF and pIRES2-revR in Figure 1A) for seamless cloning of the pCMV-DsRed-MCS-IRES-EGFP-SV40 pA with the DsRed ORF product (top half in Figure 1A) and the psiCHECK-2 plasmid using the primer pair psiCHECK2-reverse F and psiCHECK2-reverse R (Table S1; psi-revF and psi-revR in Figure 1B) for removal of its HSV-TK promoter and seamless cloning of the pSV40-hRluc-MCS-IRES-hluc-SV40 pA with the IRES element PCR product (Figure 1B). Similarly, we reverse PCR-linearized the pCMV-DsRed-MCS-IRES-EGFP-SV40 pA construct with the primer pair pIRES2-EGFP-reverse F2 and pIRES2-EGFP-reverse R2 (Table S1; pIRES2-revF2 and pIRES2-revR2 in Figure 1A) for seamless cloning of the pTK-DsRed-MCS-IRES-EGFP-SV40 pA construct with the HSV-TK promoter product 1 (Named TK product 1 in Figure 1A, bottom half) and the pSV40-hRluc-MCS-IRES-hluc-SV40 pA construct with the primer pair psiCHECK2-reverse F2 and psiCHECK2-reverse R2 (Table S1; psi-revF2 and psi-revR2 in Figure 1B) for seamless cloning of the pTK-hRluc-MCS-IRES-hluc-SV40 pA construct with the HSV-TK promoter product 2 (Named TK product 2 in Figure 1B, bottom half).

PCR amplifications of the four inserts (DsRed ORF, IRES, HSV-TK product 1, and HSV-TK product 2) and reverse PCR linearization of the 2 plasmids (pIRES2-EGFP and psiCHECK-2) and 2 bicistronic reporter vectors (pCMV-DsRed-MCS-IRES-EGFP-SV40 pA and pSV40-hRluc-MCS-IRES-hluc-SV40 pA) mentioned above were conducted individually in a 50-μL reaction containing 10 μL 5× PrimeSTAR GXL Buffer, 1 μL PrimeSTAR GXL DNA Polymerase (Takara, Dalian, China), 5 μL dNTP Mixture (2.5 mM each), 2 μL forward primer, 2 μL reverse primer, 1 μL plasmid DNA template (10 ng), and 39 μL ddH2O. These PCR or reverse PCR reactions were initiated at 95 °C for 5 min, followed by 30 cycles of 98 °C for 10 s, 60 °C for 15 s (4 insert PCRs) or for 1 min per kb (4 linearization PCRs), and a final extension of 10 min at 68 °C. The four bicistronic reporter vectors were then constructed by assembling 1 μL (50 ng) of each linearized plasmid or vector together with 1 μL (25 ng) of the corresponding insert at 50 °C for 15 min in a tube containing 5 μL 2× Seamless Master mix (Taihe Biotechnology) and 3 μL ddH2O. Five μL of each assembling reactions were transformed into DH5α *E. coli* competent cells and the resulted positive clones were verified by sequencing at Beijing Genomic Institute (BGI) (Beijing, China).

### 4.2. Generation of Synthetic pA Site (SPA) Mutant Constructs

To construct fluorescence SPA or fluorescence SPA mutant constructs, we annealed and extended 1 common reverse oligo (Red-GFP-PAS-R) with one of the 5 forward oligos that contain AATAAA (Red-GFP-PAS-wild-F), ANTAAA (Red-GFP-PAS-mut1-F), NANAAN (Red-GFP-PAS-mut3-F), NNNNNN (Red-GFP-PAS-rad-F), or no hexamer (Red-GFP-PAS-del-F) (Table S1) to form 5 different double-stranded SPA fragments. Each annealing/extension reaction (50 μL) contained 5 μL 10× ExTaq Buffer, 1 μL ExTaq DNA Polymerase (Takara), 5 μL dNTP, 5 μL forward oligo, 5 μL reverse oligo, and 29 μL ddH2O. Each annealing/extension reaction was initiated at 95 °C for 10 min, followed by 60 °C for 5 min, 72 °C for 10 min, and stored at −20 °C before cloning. We then subcloned the 5 SPA fragments into the pCMV-DsRed-MCS-IRES-EGFP-SV40 pA vector through its *EcoRI* and *BamHI* sites (Figure 4A). Likewise, we annealed and extended the common reverse oligo PSI-R with PSI-wild-F (AATAAA), PSI-del-F (No hexamer), PSI-mut1-F (ANTAAA), PSI-mut3-F (NANAAN) or PSI-rad-F (NNNNNN) (Table S1) and inserted the formed double-stranded SPA fragments into the pSV40-hRluc-IRES-hluc-SV40 pA vector through its *XhoI* and *EcoRI* sites for generation of luciferase SPA or SPA mutant constructs (Figure 5A). All the obtained fluorescence and luciferase SPA/SPA mutant construct clones were confirmed by sequencing at BGI.

### 4.3. Generation of Human CD47 pA Site Constructs

Genomic DNA was extracted from Hela cells and used as the common template for PCR amplification of the proximal (pA1) and distal (pA2) pA sites (200 bp up- and down-stream of the corresponding cleavage site) of the human *CD47* gene (Figure 6A) with the same set up and cycling conditions described above. Because both pA1 and pA2 sites were inserted either upstream of IRES or downstream of EGFP in the fluorescence vector or hRluc in the luciferase vector, we conducted 4 PCRs with 4 pairs of primers for each of the two pA sites to construct a total of 8 CD47 pA site constructs (Figure 6B). The primer pair CD47PA1-F-*EcoRI* and CD47PA1-R-*BamHI* (Table S1) were used to yield a common pA1 PCR product for construction of pCMV-DsRed-CD47 pA1-IRES-EGFP-SV40 pA and pCM-DsRed-CD47 pA1-IRES-EGFP-CD47 pA2, whereas the primer pair CD47PA1-F-*NotI* and CD47PA1-R-*AflII* were used to produce a pA1 PCR product for construction of pCMV-DsRed-CD47 pA2-IRES-EGFP-CD47 pA1 (Figure 6B). Likewise, the primer pair CD47PA2-F-*EcoRI* and CD47PA2-R-*BamHI* were used to obtain a common pA2 PCR product for construction of pCMV-DsRed-CD47 pA2-IRES-EGFP-SV40 pA and pCMV-DsRed-CD47 pA2-IRES-EGFP-CD47 pA1, while the primer pair CD47PA2-F-*NotI* and CD47PA2-R-*AflII* were employed to product a pA2 PCR product for generation of pCMV-DsRed-CD47 pA1-IRES-EGFP-CD47 pA2.

In parallel to construction of the 4 fluorescence CD47 pA site constructs, the primer pair CD47PA1-F-*XhoI* and CD47PA1-R-*EcoRI* (Table S1) were used to build pSV40-hRluc-CD47 pA1-IRES-hluc-SV40 pA and pSV40-hRluc-CD47 pA1-IRES-hluc-CD47 pA2, while the primer pair CD47PA1-F-*XbaI* and CD47PA1-R-*BamHI* were utilized to construct pSV40-hRluc-CD47 pA2-IRES-hluc-CD47 pA1 (Figure 6B). Similarly, the primer pair CD47PA2-F-*XhoI* and CD47PA2-R-*EcoRI* was designed to make pSV40-hRluc-CD47 pA2-IRES-hluc-SV40 pA and pSV40-hRluc-CD47 pA2-IRES-hluc-CD47 pA1, whereas the primer pair CD47PA2-F-*XbaI* and CD47PA2-R-*BamHI* was employed to construct pSV40-hRluc-CD47 pA1-IRES-hluc-CD47 pA2.

### 4.4. Cell Culture and Transient Transfection

Both HEK293 cells and Hela cells were routinely cultured at 37 °C with 5% CO_2_ in Dulbecco’s Modified Eagle’s (DMEM) high-glucose medium (Gibco, Grand Island, NY, USA) supplemented with 10% fetal bovine serum (Gibco) and 1% penicillin/streptomycin (HyClone, Thermo Scientific, Logan, UT, USA). For transient transfections, cells seeded onto a 6-well (for the fluorescence vector and constructs) or 12-well (for the luciferase vector or constructs) plate (1 × 10^6^ cells/mL) for 24 h were transfected with a wildtype bicistronic vector or its construct (1 μg/well) using X-tremeGENE HP DNA Transfection Reagent (2 μL/well; Roche Applied Science, Indianapolis, IN, USA), following the manufacturer’s instructions. Twelve hours post-transfection, we replaced the old culture medium with fresh medium and kept the plates in a CO_2_ incubator for another 48 h. At least three independent transfections (3 biological replicates) were conducted for each wildtype vector or construct.

### 4.5. Fluorescence Microscopy Analysis

After Hela (SPA construct and wildtype vector in Figure 3) or HEK293 cells were transfected with fluorescence SPA, SPA mutant or CD47 pA site constructs for 60 h, bright-field images of 3 selected fields of view for every well of each plate were acquired at 100 or 200 magnification under a fluorescence microscope IX83 (Olympus, Tokyo, Japan). We further acquired the EGFP and DsRed fluorescence images of the same 3 fields of view of each well by exposing the cells for 50 milliseconds (ms) with the GFP filter set (excitation/emission, 470/520 nm) and for 200 ms with the DsRed filter set (excitation/emission, 535/565 nm), respectively. The acquired images were scanned and analyzed to measure the DsRed or EGFP fluorescence intensity in the cells of the entire field of view using the Image J version 1.46 software (National Institutes of Health, Bethesda, MD, USA). At least 3 independent transfections were conducted for each construct or wildtype vector (control); and images of 3 different fields of view were acquired and analyzed for each of the 3 transfections per construct or wildtype vector (i.e., *n* = 3 × 3 = 9). The DsRed and EGFP fluorescence intensities of each construct were normalized by dividing them by the average DsRed and EGFP fluorescence intensities of the wildtype vector, respectively. The average normalized EGFP/DsRed intensity ratio of at least 3 independent transfections per construct or wildtype vector was calculated to represent the relative expression of EGFP and DsRed at the protein level.

### 4.6. Fluorescent Cell Flow Cytometry Analysis

HEK293 cells seeded onto 6-well plates at 1 × 10^6^ cells/mL and transfected with fluorescence SPA or SPA mutant constructs were gently suspended by trypsin (0.25%, Hyclone, 0.5 mL/well and digestion for 1 min in room temperature) 60 h post-transfection. The suspended cells were then subjected to flow cytometry analysis using the MoFlo XDP cell sorter (Beckman Coulter, Brea, CA, USA). The cells were excited by a 488 nm laser beam and the emitted EGFP and DsRed fluorescence signals were captured at 532 nm in the detector FL1 and at 561 nm in the detector FL2, respectively. We used the HEK293 cells co-transfected with pIRES2-EGFP and pDsRed-N1 to adjust the green or red fluorescence signals in the FL1 and FL2 channels, respectively. The slopes of the resulted signal plots of the two channels were used to indicate the relative expression of EGFP and DsRed at the protein level.

### 4.7. Dual Luciferase Activity Assay

Hela (SPA construct and wildtype vector in Figure 3) or HEK293 cells transfected with the wildtype luciferase vector or its construct were harvested 60 h post-transfection. The resulting lysates were used to measure the humanized renilla (hRluc) and firefly (hluc) luciferase activities with the Dual-Luciferase Reporter Assay kit (Promega, Madison, WI, USA) on a luminometer (GloMax 20/20, Promega) according to the manufacturer’s instructions. At least three independent transfections (3 biological replicates) were conducted for each construct or the wildtype vector. The hRluc and hluc activities of each construct were normalized with the average hRluc and hluc activities of the wildtype luciferase reporter vector, respectively. The average normalized hluc/hRluc luciferase activity ratio of at least 3 independent transfections per construct or wildtype vector was calculated to represent the relative expression of the two luciferases at the protein level.

### 4.8. Quantitative RT-PCR (qRT-PCR) Analyses of Fluorescence and Luciferase Reporter Genes

Total RNAs were extracted from HEK293 or Hela cells transfected with the fluorescence or luciferase vector/construct with Trizol reagent (Thermo Fisher Scientific, Waltham, MA, USA) and then treated with DNase I (Promega) and RNase inhibitor (Thermo Fisher Scientific) for 40 min to remove potential genomic DNA (gDNA). One µg of each total RNA sample was reverse transcribed into cDNA at 42 °C for 0.5 h in a 20 µL reaction with 2 µL 6-mer random primer, 2 µL dNTP (2.5 mM, Takara), 1 µL M-MuLV reverse transcriptase (New England Biolab, Boston, MA, USA), and 1 µL RNase inhibitor (Thermo Fisher Scientific). The cDNAs were diluted 10-fold and used as the templates for qRT-PCR analyses of *EGFP, DsRed*, and *Kana* resistance gene (internal reference gene) for the fluorescence vector and constructs as well as *hluc*, *hRluc*, and *Amp* resistance gene (internal reference gene) for the luciferase vector or constructs. The primer pairs for qRT-PCR analyses of the 4 target (*DsRed*, *EGFP*, *hluc*, *hRluc*) and 2 reference genes (*Kana* and *Amp* resistance genes) and their amplification efficiencies are summarized in Table S2. 

qPCRs of *DsRed*, *EGFP*, *hluc*, *hRluc*, *Kana* and *Amp* were individually performed in a 20 µL reaction containing 1 µL 10 fold-diluted cDNA, 0.5 µL of each gene-specific forward and reverse primers (Table S2; 10 µM each), 10 µL 2× SuperReal PreMix Plus (Tiangen Biotech, Beijing, China), 0.4 µL 50 × ROX Reference Dye (Tiangen biotech), and 7.6 µL ddH2O with an ABI 7500 Real-Time PCR System (Applied Biosystems, Foster City, CA, USA). The cycling program was composed of an initial denaturation at 95 °C for 15 min, followed by 40 cycles of denaturation at 95 °C for 15 s, annealing at 60 °C for 30 s and extension at 72 °C for 32 s, during which real-time data were collected. Melting curve analysis was performed from 65 to 95 °C for all the target and reference genes to ensure they were free of junk products. Each biological replicate was qRT-PCR-analyzed in triplicate. Amplification efficiency (E) of each gene was determined from the slope of the log template concentration (*x*-axis)-*C*t value (*y*-axis) line, using the formula E = 10^−1/slope^ − 1 [43]. The normalized expressions of the four target genes were calculated by the formula of 2^−ΔΔ*C*t^. We further divided the normalized expression of *EGFP* or *hluc* by that of *DsRed* or *hRluc* to indicate the relative expression of the two fluorescence or luciferase reporter genes at the mRNA level.

## 5. Data Analysis

One-way analysis of variance (ANOVA) followed by Tukey’s HSD tests were performed to test the significance of differences in the relative expression levels of EGFP and DsRed or hluc and hRluc at the protein (fluorescence intensity or luciferase activity ratios) and mRNA (qRT-PCR data) levels among different fluorescence or luciferase constructs. When there were only two treatments (e.g., wildtype vector vs. synthetic pA site construct in Figure 3), we conducted independent *t*-tests to compare the significance of differences between the two treatments. All of the statistical tests were completed by SPSS version 19.0 software (SPSS Inc., Chicago, IL, USA) and GraphPad Prism 5.0 software (GraphPad Software Inc., San Diego, CA, USA).

## Supplementary Materials

Supplementary materials can be found at www.mdpi.com/1422-0067/19/1/279/s1.
