# Supplementary material for: Useful Bicistronic Reporter System for Studying Poly(A) Site-Defining cis Elements and Regulation of Alternative Polyadenylation
Source: Int J Mol Sci. 2018 Jan 17;19(1):279. doi: 10.3390/ijms19010279 (PMC5796225; doi:10.3390/ijms19010279)
Supplement: Supplementary file 1 [file ijms-19-00279-s001.pdf]

Supplementary Materials

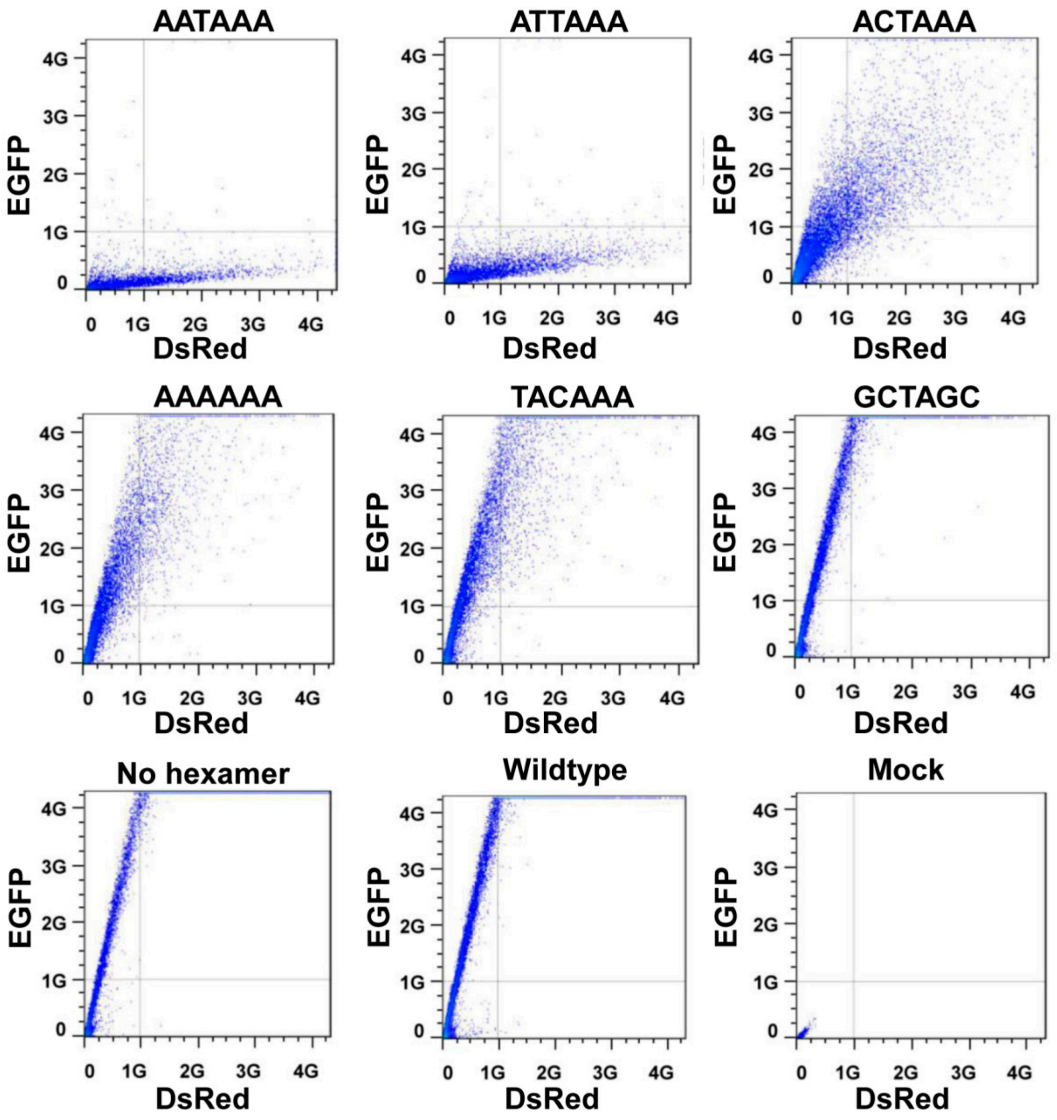

Figure S1. Fluorescent activated cell sorting (FACS) of HEK293 cells transfected with wildtype or mutant SPA constructs with different hexamer variants. HEK293 cells determined to be expressing both red and green were gated in the untransfected sample. The Y axis represents intensity of green fluorescence (EGFP) and the X axis represents the intensity of red fluorescence (DsRed). The slope represents the EGFP/DsRed radio.

CD47-pA1 (200bp up-and downstream of the cleavage site)

AGTGAAGTGATGGACTCCGATTTGGAGAGTAGTAAGACGTGAAAGGAAT
ACACTTGTGTTTAAGCACCATGGCCTTGATGATTCACTGTTGGGGAGAAG
AAACAAGAAAAGTAACTGGTTGTCACCTATGAGACCCTTACGTGATTGTT
AGTTAAGTTTTTATTCAAAGCAGCTGTAATTTAGTTAATAAAATAATTATG
ATCTATGTTGTTTGCCCAATTGAGATCCAGTTTTTTGTTGTTATTTTTAATC
AATTAGGGGCAATAGTAGAATGGACAATTTCCAAGAATGATGCCTTTCAG
GTCCTAGGGCCTCTGGCCTCTAGGTAACCAGTTTAAATTGGTTCAGGGTGA
TAACTACTTAGCACTGCCCTGGTGATTACCCAGAGATATCTATGAAAACC
AGTGG

CD47-pA2 (200bp up-and downstream of the cleavage site)

CATTTTAAGCTATTTTGTTGGGCTATTTCTATTGCTGCTACAGCAGACCAC
AAGCACATTTCTGAAAAATTTAATTTATTAATGTATTTTTAAGTTGCTTAT
ATTCTAGGTAACAATGTAAAGAATGATTTAAAATATTAATTATGAATTTTT
TGAGTATAATACCCAATAAGCTTTTAATTAGAGCAGAGTTTTAATTAAAA
GTTTTAAATCAGTCCAATGTGTTTCATAATTATTTTTTAATTTTCTCCATGG
TGCAGTGAAAACATCACATGAGAAAGCAGGAGACCTGGGTGCTAGTCCTG
GCTACACTACCAGCTAGCAGAGGACCTGGGAAAGCTACTTCCCTGGATCT
CAGTGTCCGTCTCTGCCTGTAGAATGAGATAAGTCTCTTCAAGACTGAAAT
GTATGGTTCTCCATTTTAATTATCAG

Figure S2. The sequences and *cis*-acting elements of the two pA sites of human CD47 gene. Yellow = AAUAAA or AAUAAA like hexamer, Arrowhead = cleavage site,
Blue = downstream U/GU-rich element. Green = upstream auxiliary elements UGUA and U–rich element.

Table S1. Oligos used for construction of the wildtype reporter vectors and various constructs.

Oligo name	Sequence (5′ to 3′) [a]
Oligos for construction of the 4 wildtype dicistronic vectors	
DsRed-homology F	*TCTCGAGCTCAAGCTTC*ATGGACAACACCGAGGACGTCATCA
DsRed-homology R	*TGAGTCCGGACTGGGA*GCCGGAGTGGCGGGCCTCGGCGTGCTC
IRES-homology F	*GTTTTTTGTGTGAGGATCT*GAATTCGCCCCTCTCCCTCCCCCCCCCCTAA
IRES-homology R	*CTTAGCATCGGCCATGGT*TGTGGCCATATTATCATCGTGTTTTTC
pTK-homology F1	CGGA*AGATCTAAATGAGTCTTCGGAC*CTCGCG
pTK-homology R1	GGCA*GCTAGCGTGGCTTTACCAACA*GTACCGGAAT
pTK-homology F2	*AGATCTAAATGAGTCTTCGGAC*CTCGCG
pTK-homology R2	*GCTAGCGTGGCTTTACCAACA*GTACCGGAAT
pIRES2-EGFP-reverse F	*TCCCAGTCCGGACTCA*GATCTCGAGCTCAA
pIRES2-EGFP-reverse R	*GAAGCTTGAGCTCGAGA*TCTGAGTCCGGTAG
psiCHECK2-reverse F	*ACCATGGCCGATGCTAAG*AACATTA
psiCHECK2-reverse R	*AGATCCTCACACAAAAAAC*CAACACAC
pIRES2-EGFPreverse F2	*TGTTGGTAAAGCCACGCTAGC*GCTACCGGACTCAGATCTCGAGCT
pIRES2-EGFPreverse R2	*GTCCGAAGACTCATTTAGATCT*GACCCCGTAATTGATTACTATTAATAACTA
psiCHECK2-reverse F2	*TGTTGGTAAAGCCACGCTAGC*CACCATGGCTTCCAAGGTGTACGACCCCGAGC
psiCHECK2-reverse R2	*GTCCGAAGACTCATTTAGATCT*GTCGAGCCATGTGAGCAAAAGGCCAGCAAAAG
Oligos for construction of SPA or SPA mutant constructs	

Red-GFP-PAS-wild-F	CTGCGGAATTCAATAAAATATCTTTATTTTCATTACATCTGTGTGTTGG
Red-GFP-PAS-R	CTGCGGGATCCTCACACAAAAAACCAACACACAGATGTAATGAAAAT
PSI-wild-F	CTCCGCTCGAGAATAAAATATCTTTATTTTCATTACATCTGTGTGTTGG
PSI- R	CTGCGGAATTCTCACACAAAAAACCAACACACAGATGTAATGAAAAT
Red-GFP-PAS-mut1-F	CTGCGGAATTCANTAAAATATCTTTATTTTCATTACATCTGTGTGTTGG
Red-GFP-PAS-mut3-F	CTGCGGAATTCNANAANATATCTTTATTTTCATTACATCTGTGTGTTGG
Red-GFP-PAS-rad-F	CTGCGGAATTCNNNNNNATATCTTTATTTTCATTACATCTGTGTGTTGG
Red-GFP-PAS-del-F	CTGCGGAATTCATATCTTTATTTTCATTACATCTGTGTGTTGG
PSI-mut1-F	CTCCGCTCGAGANTAAAATATCTTTATTTTCATTACATCTGTGTGTTGG
PSI-mut3-F	CTCCGCTCGAGNANAANATATCTTTATTTTCATTACATCTGTGTGTTGG
PSI-rad-F	CTCCGCTCGAGNNNNNNATATCTTTATTTTCATTACATCTGTGTGTTGG
PSI-del-F	CTCCGCTCGAGATATCTTTATTTTCATTACATCTGTGTGTTGG
Oligos for construction of CD47 pA site constructs	
CD47PA1-F-*EcoRI*	GCGAGGAATTCAGTGAAGTGATGGACTCCGATT
CD47PA1-R-*BamHI*	GCGAGGGATCCCCACTGGTTTTCATAGATATCTC
CD47PA1-F-*XhoI*	GCGAGCTCGAGAGTGAAGTGATGGACTCCGATT
CD47PA1-R-*EcoRI*	GCGAGGAATTCCCACTGGTTTTCATAGATATCTC
CD47PA1-F-*NotI*	AAGGAAAAAAGCGGCCGCAGTGAAGTGATGGACTCCGATT
CD47PA1-R-*AflII*	GCGAGCTTAAGCCACTGGTTTTCATAGATATCTC
CD47PA1-F-*XbaI*	GCGAGTCTAGAAGTGAAGTGATGGACTCCGATT
CD47PA2-F-*EcoRI*	GCGAGGAATTCCATTTTAAGCTATTTTGTTGGGC
CD47PA2-R-*BamHI*	GCGAGGGATCCCTGATAATTAAAATGGAGAACC
CD47PA2-F-*XhoI*	GCGAGCTCGAGCATTTTAAGCTATTTTGTTGGGC
CD47PA2-R-*EcoRI*	GCGAGGAATTCCTGATAATTAAAATGGAGAACC
CD47PA2-F-*NotI*	AAGGAAAAAAGCGGCCGCCATTTTAAGCTATTTTGTTGGGC
CD47PA2-R-*AflII*	GCGAGCTTAAGCTGATAATTAAAATGGAGAACC
CD47PA2-F-*XbaI*	GCGAGTCTAGACATTTTAAGCTATTTTGTTGGGC

[a] The italic letters represent the shared homology sequences between the corresponding insert and the corresponding linearized plasmids or vectors.

Table S2. Primer pairs used for qRT-PCR analysis of the reporter or reference genes.

Gene name (abbreviation)	Sequence (5′ to 3′) [a]	Product length (bp)	Primer efficiency (%)	R^2 [b]
DsRed	F:GCCCCGTAATGCAGAAGAAG	101	99.37	0.9999
DsRed	R:CTTCAGGGCGTGGGAGATCT			
EGFP	F:GGGCACAAGCTGGAGTACAACT	101	100.11	0.9991
EGFP	R:ATGTTGTGGCGGATCTTGAAG			
Kana	F:GCCGAATATCATGGTGGAAA	106	95.55	0.9998
Kana	R:AATATCACGGGTAGCCAACG			
Hluc	F:CGTGCCAGAGTCTTTCGACA	106	101.05	0.9997
Hluc	R:ACAGGCGGTGCGATGAG			
HRluc	F:AAGAGCGAAGAGGGCGAGAA	217	95.96	0.9995
HRluc	R:TGCGGACAATCTGGACGAC			
Amp	F:ACTCGGTCGCCGCATACACTA	178	103.30	0.9999
Amp	R:GGTTAGCTCCTTCGGTCCTCC			

[a] F and R respectively indicate forward primer and reverse primer. [b] R^2 refers to the coefficient of determination.
